# Supplementary material for: Identification of photoperiod-induced specific miRNAs in the adrenal glands of Sunite sheep (Ovis aries)
Source: Front Vet Sci. 2022 Jul 22;9:888207. doi: 10.3389/fvets.2022.888207 (PMC9354845; doi:10.3389/fvets.2022.888207)
Supplement: Supplementary file 1 [file Data_Sheet_1.ZIP › Supplementary file/miRDeep2 file.zip!/miRDeep2 file/Novel_Structure/SPLP42A3/Novel_544.pdf]

Provisional ID	: NC_019460.2_10724
Score total	: 2.3
Score for star read(s)	: -1.3
Score for read counts	: 0
Score for mfe	: 2
Score for randfold	: 1.6
Score for cons. seed	:
Total read count	: 16
Mature read count	: 12
Loop read count	: 0
Star read count	: 4

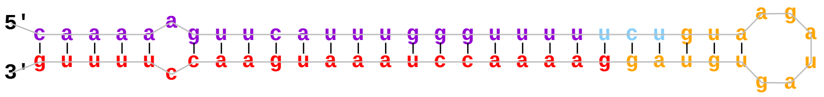

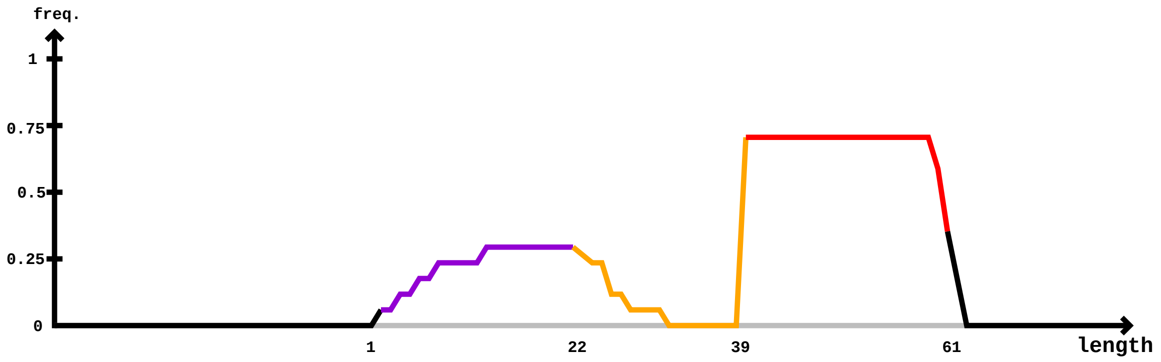

Star Mature

```
5'- uuugugccuggguguuaauuuauugagugggccaaaaaguucauuuggguuuuucuguaagauaguguaggaaaaccuaaaugaaccuuuuggccagcccaacacaaagg  -3'  obs
    uuugugccuggguguuaauuuauugagugggccaaaaaguucauuuggguuuuucuguaagauaguguaggaaaaccuaaaugaaccuuuuggccagcccaacacaaagg        exp
    ((((((..(((((................((((((((.(((((((((((((((((((((......))))))))))))))))))))).)))))))).))))).))))))..  reads  mm  sample
    ................................caaaaaguucauuugCguuuu.........................................................  1  1  seq
    ..................................aaaaguucauuAggguuuuucu......................................................  1  1  seq
    ....................................aaguucauuuggguuuuucC......................................................  1  1  seq
    ......................................guucauuugggGuuuucugu....................................................  1  1  seq
    ...........................................uuugggGuuuucuguaaga................................................  1  1  seq
    ......................................................................gaaaaccuGaaugaaccuuu....................  1  1  seq
    ......................................................................gaaaaccuaaaugaaccuuu....................  1  0  seq
    ......................................................................gaaaaccuGaaugaaccuuuu...................  4  1  seq
    ......................................................................gaaaaccuGaaugaaccuuuug..................  6  1  seq
```
